# Prevalence and Seroprevalence of *Trypanosoma cruzi* Infection in a Military Population in Texas

**DOI:** 10.4269/ajtmh.17-0109

**Published:** 2017-08-14

**Authors:** Bryant J. Webber, Mary T. Pawlak, Sandra Valtier, Candelaria C. Daniels, Charla C. Tully, Edward J. Wozniak, Walter D. Roachell, Francisco X. Sanchez, Audra A. Blasi, Thomas L. Cropper

**Affiliations:** 1 59th Medical Wing, Joint Base San Antonio, Lackland, San Antonio, Texas;; 2 Brooke Army Medical Center, Joint Base San Antonio, Fort Sam Houston, San Antonio, Texas;; 3 Landstuhl Regional Medical Center, Landstuhl, Germany;; 4 Texas State Guard Medical Brigade Headquarters, Camp Mabry, Austin, Texas;; 5 US Army Public Health Command Central, Joint Base San Antonio, Fort Sam Houston, San Antonio, Texas

## Abstract

Recent biosurveillance findings at Joint Base San Antonio (JBSA), a large military installation located in south-central Texas, indicate the potential for vector-borne human Chagas disease. A cross-sectional study was conducted to determine the prevalence and seroprevalence of *Trypanosoma cruzi* infection in highest risk subpopulations on the installation, including students and instructors who work and sleep in triatomine-endemic field settings. Real-time polymerase chain reaction, enzyme-linked immunosorbent assay, and indirect immunofluorescent antibody assay were performed on enrolled subjects (*N* = 1,033), none of whom tested positive for *T. cruzi* or anti-*T. cruzi* antibodies. Current countermeasures used during field training on JBSA appear to be sufficient for preventing autochthonous human Chagas disease.

## INTRODUCTION

Chagas disease, or American trypanosomiasis, is caused by infection with *Trypanosoma cruzi*. The protozoan parasite is transmitted to humans most commonly through the infected excreta of hematophagous triatomine insects of the family Reduviidae, entering the bloodstream through a wound or mucous membrane.^1^ Known colloquially as “kissing bugs,” triatomines are found throughout the western hemisphere. Eleven species are endemic to the southern United States, most of which are competent vectors of the parasite.^2^ In addition to the vector-borne route, *T. cruzi* may be transmitted congenitally, orally in contaminated food or beverages, or directly via blood transfusion or organ and tissue transplantation.^3^

The majority of human cases are subclinical in both the acute and chronic stages, resulting in lifelong, undiagnosed infection. Cardiac disease, gastrointestinal disease, or both develop in approximately one-third of cases, typically manifesting years or decades after initial infection.^4^ Chagas disease mortality is usually attributed to heart failure or ventricular arrhythmia,^5^ but even asymptomatic infection with *T. cruzi* may increase all-cause mortality risk.^6^

Between 5 and 8 million people globally are infected with *T. cruzi*,^1,7^ incurring an annual economic burden of 7.2 billion USD.^8^ Although vectorial transmission is restricted to the Americas,^9^ human migration from endemic to nonendemic countries—and within endemic countries from rural to urban areas—has broadened the distribution of prevalent Chagas disease.^10^ Both imported and autochthonous cases occur in the United States, with the former predominating: at least 240 thousand Latin American immigrants are presumably infected,^11^ whereas fewer than 30 locally acquired infections have ever been reported.^12^ The dearth of documented autochthonous cases, however, may be more indicative of provider unawareness and suboptimal surveillance than true disease incidence.^12,13^ Recognizing the potential for vector-borne transmission across a broad swath of the southern United States, the Centers for Disease Control and Prevention (CDC) prioritizes Chagas as one of five neglected parasitic infections^14^ and urges more research to define autochthonous infection risk.^12^

In the greater San Antonio metropolitan area and throughout south-central Texas, Chagas disease has been a known but vaguely defined human disease threat since at least the 1960s.^15–17^ An obligation to elucidate that threat recently emerged from several biosurveillance findings on Joint Base San Antonio (JBSA), one of the largest military training installations in the United States. A 2007 serosurvey of military working dogs, all of which are trained on JBSA, found that 24 (8%) harbored anti-*T. cruzi* antibodies. Multiple military working dogs serving in Iraq required evacuation because of cardiomyopathy, later attributed to Chagas disease.^18^ A faunal survey was commissioned, which found that 43% (88/205) of collected adult triatomines and 22% (163/736) of nymphs tested positive for *T. cruzi* on polymerase chain reaction (PCR), and blood meal analysis revealed that 33% (43/131) contained human blood in their midgut. Among adults, *Triatoma sanguisuga* (66%) and *Triatoma gerstaeckeri* (30%) were the most common species identified (C. Daniels, unpublished data). This prompted the enforcement of new administrative, technical, and personal protective measures—as well as the reinforcement of existent measures—to protect humans and dogs against vector-borne pathogen exposure during field exercises on JBSA. Because triatomines were collected inside tents and elsewhere near populous training sites, we initiated this study to determine human infection risk.

## MATERIALS AND METHODS

This cross-sectional study was designed to establish the prevalence of *T. cruzi* parasitemia and seroprevalence of anti-*T. cruzi* antibodies in five subpopulations most at risk for vector-borne infection while training and working on the installation: students graduating from the US Air Force Security Forces Apprentice course, all of whom had completed a week-long field training exercise on a triatomine-endemic site of the installation at least 15 weeks before study enrollment; instructors from the US Air Force Security Forces Apprentice course; instructors from the US Air Force Basic Military Training field training course; instructors from the Department of Defense Military Working Dog school; and instructors from the US Air Force Survival, Evasion, Resistance, and Escape course. Given reduced prevalence of triatomines during the winter months,^19^ and thus reduced likelihood of detecting parasitemia and anti-*T. cruzi* IgM antibodies, we suspended enrollment from December through March.

We administered a questionnaire to all consented participants to gather demographic data, quantify exposure risk, and ascertain the geographic location of infection, should a subject test positive. Demographic data included age, sex, and self-reported race and ethnicity. The questionnaire initially focused on vectorial transmission risk by extracting information regarding military training; permanent residence in and travel to triatomine-endemic areas^1,2^; camping, hunting,^20^ and exposure to reservoir wildlife^3,17^ in triatomine-endemic areas; and bites by triatomines or by unidentified insects that may have been triatomines. We displayed high-resolution photos of *T. sanguisuga* and *T. gerstaeckeri* species to facilitate an accurate bite history. After discussing preliminary results with two external consultants, we added questions pertaining to blood transfusion and congenital transmission routes.

We collected whole blood from consented volunteers by peripheral venipuncture. On all subjects from whom we could obtain sufficient aliquots, we performed real-time PCR to determine the prevalence of *T. cruzi* parasitemia and an enzyme-linked immunosorbent assay (ELISA) and indirect immunofluorescent antibody (IFA) test to determine the seroprevalence of anti-*T. cruzi* antibodies.

Real-time PCR was conducted in singlicate per CDC published methodology to detect *T. cruzi* DNA in human samples.^21^ Briefly, DNA was extracted from buffy coat fractions from EDTA whole blood specimens followed by two multitarget TaqMan real-time PCR assays targeting three highly conserved and repetitive *T. cruzi* genomic regions: nuclear minisatellite TCZ; kinetoplast DNA; and the small subunit ribosomal RNA (18S rRNA) gene. Internal amplification control was performed with the RNase P human gene from the TaqMan Human RNase P detection reagent (Applied Biosystems, Foster City, CA).^22^ A blood sample was considered positive if it was PCR-positive for all three *T. cruzi* targets and equivocal if it was PCR-positive for only two *T. cruzi* targets. Equivocal results prompted retesting.

The Chagatest ELISA recombinante v.3.0 (Wiener Laboratórios, Rosario, Argentina) was performed in duplicate, as directed by the manufacturer, for detecting human IgG and IgM anti-*T. cruzi* antibodies. Collected serum samples were incubated with immobilized antigen, washed, incubated with goat antihuman IgG conjugated to horse radish peroxidase, and washed again. Tetramethylbenzidine and hydrogen peroxide were then added, and the reactions were stopped with 2 N sulfuric acid. The colorimetric readings were taken at 450 nm in a plate reader, using a reference wavelength of 650 nm. Cutoff values were defined, per manufacturer guidance, as the mean negative control readings plus 0.3. The test was considered positive if greater than the cutoff value plus 10%, negative if less than the cutoff value minus 10%, and equivocal if within ± 10% of the cutoff value.

Sera were also evaluated for the presence of human IgG antibodies against *T. cruzi* via IFA technique. A positive control and negative control were validated from the 21-member panel of the Chagas Titer AccuSet^™^ Performance Panel (SeraCare Life Sciences, Milford, MA). Panel member 1, which had an antibody titer of 1:4,096, was used as a positive control at a 1:200 dilution. Panel member 21, which had a negative titer (< 1:128), was used as a negative control, also at a 1:200 dilution. Subject samples were screened at a 1:128 dilution. The dilutions were placed on *T. cruzi* antigen–coated microscope slides, washed to remove unbound serum antibodies, stained with a fluorescein isothiocyanate–labeled goat antihuman IgG conjugate, and visualized through a fluorescence microscope. A sample was considered positive at a titer equal to or greater than 1:128. IFA testing was conducted in singlicate.

We used descriptive statistics to build demographic and exposure profiles of the enrolled sample, both collectively and stratified by student and instructor status. We compared exposure time between students and instructors with an unpaired *t* test, using Epi Info v7.0 (CDC, Atlanta, GA). This study was approved by the 59th Medical Wing Institutional Review Board (FWH20140074H). Written informed consent was obtained from all subjects.

## RESULTS

A total of 1,033 subjects were enrolled. Consistent with the ratio of students to instructors on the installation, the vast majority of participants (93.1%) were students graduating from the Security Forces Apprentice course (Table 1). During the 16-month study period (April–November 2015 and April–November 2016), we enrolled approximately 15% and 30% of eligible students and instructors, respectively. Most subjects were male (76.9%) and white, non-Hispanic (54.8%). The mean (standard deviation [SD]) age was 21.6 (4.6) years. Three subjects experienced presyncope with venipuncture, all of whom recovered fully without medical intervention. No other adverse events were noted.

**Table 1:** Demographic and risk factor profile of study subjects

	Students, *N* = 962	Instructors, *N* = 71	Total, *N* = 1,033
Population			
Security forces apprentice students	962 (100%)	–	962 (93.1%)
Security forces apprentice instructors	–	2 (2.8%)	2 (0.2%)
Basic military training field training instructors	–	36 (50.7%)	36 (3.5%)
Military working dog school instructors	–	23 (32.4%)	23 (2.2%)
Survival, evasion, resistance, and escape instructors	–	10 (14.1%)	10 (1.0%)
Age, mean (SD)	20.9 (3.6)	31.6 (5.0)	21.6 (4.6)
Sex			
Male	735 (76.4%)	59 (83.1%)	794 (76.9%)
Female	227 (23.6%)	12 (16.9%)	239 (23.1%)
Race/ethnicity			
White, non-Hispanic	515 (53.5%)	51 (71.8%)	566 (54.8%)
Black, non-Hispanic	121 (12.6%)	7 (9.9%)	128 (12.4%)
Hispanic	221 (23.0%)	8 (11.3%)	229 (22.2%)
Other	105 (10.9%)	5 (7.0%)	110 (10.6%)
Potential exposures			
Known triatomine bite	4 (0.4%)	1 (1.4%)	5 (0.5%)
Unidentified insect bite	102 (10.6%)	29 (40.8%)	131 (12.7%)
Received blood products in the United States*	7 (0.8%)	3 (5.9%)	10 (1.1%)
Received blood products outside the United States*	3 (0.3%)	0	3 (0.3%)
Mother from endemic country*	90 (10.2%)	4 (7.8%)	94 (10.1%)
Weeks in triatomine-endemic area, mean (SD)			
Field environment at JBSA-Lackland	4.0 (0.4)†	47.0 (45.6)	7.7 (18.0)
Camping/hunting in Latin America or southwest United States‡	30.8 (125)	75.8 (284)	35.2 (147)
Wildlife exposure§ in Latin America or southwest United States‡	110.2 (281)	246.1 (514)	126.7 (320)
Living/traveling in Latin America	78.0 (230)	80.9 (225)	67.3 (212)
Living/traveling in southwest United States‡	430.4 (486)	238.9 (379)	380.9 (474)

JBSA = Joint Base San Antonio; SD = standard deviation.

* *N* = 931 because these questions were added to the questionnaire after study initiation.

† In addition to the 1-week field training exercise, sleeping in tents, at least 15 weeks before enrollment, most had recently completed 3 weeks of daytime field training, during which they slept in barracks.

‡ Southwest United States was defined as Arizona, California, Colorado, Nevada, New Mexico, Oklahoma, Texas, and Utah.

§ Wildlife exposure was defined as either hunting or living in a dwelling infested by woodrats, raccoons, opossums, skunks, wild hogs, coyotes, or deer.

Five subjects (0.5%) reported a triatomine bite and 131 (12.7%) reported a bite from an unidentified insect that may have been a triatomine. Subjects experienced 8,130 weeks of total exposure time in the triatomine-endemic field environment of JBSA, for a mean (SD) of 7.7 (18.0) weeks. Instructors (47.0 [45.6] weeks) had a greater mean exposure time than students (4.0 [0.4] weeks) (*P* < 0.001). Details on demography, binary risk factors, and time residing and conducting higher risk activities in triatomine-endemic areas are provided in Table 1.

All PCR (*N* = 1017), ELISA (*N* = 1023), and IFA (*N* = 1023) tests were negative, with the exception of one equivocal ELISA result. The enrollment total exceeds laboratory result figures because adequate blood specimens could not be obtained on every subject. The indeterminate ELISA result (0.279 IV [equivocal range: 0.27–0.33 IV]) belonged to a student of Hispanic ethnicity, who was born and lived in Central America for 2 years before emigration to the United States. He then lived in the southwest United States for 19 years before arrival at JBSA. During training, he experienced three bites that may have been from triatomines, although he could not definitively classify the insect. Repeat ELISA testing was also equivocal, and his PCR and IFA testing were negative. He was advised to visit his health care provider for further discussion and workup.

## DISCUSSION

Of 1,033 enrolled subjects, none tested positive for either *T. cruzi* parasites or anti-*T. cruzi* antibodies, suggesting that Chagas disease is currently a low threat for military personnel on JBSA. One subject, who tested negative on PCR and IFA, had an equivocal ELISA result. Even if he were truly infected, his case could not be conclusively categorized either as autochthonous, because of his early childhood spent in Latin America, or as vectorial, given the possibility of vertical transmission.

The apparent mismatch between these reassuring findings and the troubling biosurveillance signals—to include cases of canine Chagasic cardiomyopathy and a high volume of *T. cruzi*–infected triatomines—could have several explanations. Exposure of our study subjects to triatomines may have been limited by several anteceding countermeasures: vegetation reduction^23^ and application of pyrethroid-based insecticides^2^ around tents and field training sites; aggressive reduction of zoonotic reservoirs, particularly woodrats^24^ and feral swine^25^; requirements pertaining to field uniform wear (i.e., long pants bloused under boots and long sleeves secured at the wrist with buttons); distribution of DEET-based insect repellents free of charge; and, specifically for students during their field training exercise, sleeping in permethrin-treated bed nets.^26^

These countermeasures alone cannot explain our negative findings, however, because one-third of triatomines collected on our field training sites had fed on humans, and more than 13% of our subjects reported bites by triatomines or unidentified insects. Other protective factors may have contributed. First, the stercorarian transmission of parasite from vector to human host is inefficient. Statistical modeling estimates that one case of vectorial transmission requires 900–4,000 contacts with an infected triatomine.^27^ Second, triatomines opportunistically feed on a variety of vertebrate species—many of which are present on JBSA and have tested positive for *T. cruzi* infection in south-central Texas^28,29^—offering competitive, nonhuman blood meal sources. Third, species-specific vector competency may vary based on environmental distribution, flying and dispersal capacity, inclination to invade human dwellings, and feeding and defecation patterns.^2,30,31^ Unlike *Triatoma infestans*, *Triatoma dimidiata*, and *Rhodnius prolixus*, the predominant *T. cruzi* vectors in South America,^2,3^ the sylvatic species indigenous to the southern United States are less likely to defecate while taking a blood meal^32,33^ and to colonize domestic and peridomestic settings.^2^ These biologic and ecologic dynamics may partially explain why autochthonous human Chagas disease appears to be of relatively low risk in the United States,^12^ despite a high prevalence of *T. cruzi* infection in triatomine vectors^18,19,34–36^ and substantial evidence for triatomine feeding on humans.^34,37,38^ Risk for autochthonous human infection is associated with increased age,^39^ Hispanic ethnicity, and living in rural^40,41^ or poverty-stricken^39^ areas or near the United States–Mexico border.^29,42^ Wild and domestic animals, including military working dogs, may have an increased risk due to oral ingestion of triatomines.^29^

Another explanation, albeit a study limitation and not a causative factor, is the possibility of false-negative testing. According to a recent meta-analysis, the Weiner ELISA has a sensitivity of 93.7% (95% confidence interval: 87.7%, 96.9%) for detecting human antibodies against *T. cruzi*.^43^ IFA testing is approximately 90% sensitive, although substantial heterogeneity exists across studies.^44^ This may be due to methodological differences, particularly with titer dilution cutoffs used to determine positive results. Our 1:128 dilution screening was designed to maximize overall test accuracy, but other laboratories may demarcate a positive result at a titer of 1:80,^45^ thus conceding some specificity for improved sensitivity. PCR is rarely used in isolation to rule out disease. Although it has some diagnostic value during the parasitemic acute stage of disease,^4^ its sensitivity for detecting chronic infection in adults is below 50%.^43^ However, by using three tests in parallel and analyzing more than a thousand subjects, imperfect sensitivity for any one test does not undermine conclusions drawn this study.

The lack of molecular and serologic evidence of *T. cruzi* infection in our study sample, although encouraging, should not be misapplied. First, our findings should not be used to exclude Chagas disease from the differential diagnosis list when evaluating service members who trained or worked on triatomine-endemic field sites of JBSA. Although we selected populations with the highest risk of exposure, we only tested a sample thereof, and many had a short duration of exposure. Our results, therefore, may not be generalizable to populations with prolonged exposures or who trained on the installation before aggressive countermeasures were deployed. Second, our findings should not be used to establish countermeasure success. In the absence of a control group not employing these measures, our study was not designed to verify their effectiveness. Current countermeasures appear sufficient but not categorically necessary in preventing autochthonous human Chagas disease on JBSA.

Despite an abundance of *T. cruzi*–infected triatomine vectors, some of which evidently feed on humans, Chagas disease is currently not a major infectious disease threat for military students and instructors on JBSA. Even if parasite transmissibility is intrinsically unlikely due to bioecological factors, primary preventive measures reducing exposure to *T. cruzi* and other vector-borne pathogens should continue. To stage realistic military field training exercises while maximizing the health of humans, animals, and the environment, we urge holistic One Health approaches built on collaboration between military training leadership, civil engineers, and medical, veterinary, and public health personnel.
